# 1008. Presence of Antibody Dependent Cell Cytotoxicity (ADCC) Functional Antibodies that Target a Complex Gp41 Epitope Correlates with Long-term Non-progression and ADCC is Maintained with Mutants Using Germline Heavy Chain Variable Gene Sequence of VH1-02 Gene

**DOI:** 10.1093/ofid/ofab466.1202

**Published:** 2021-12-04

**Authors:** Sarah Baron, Meghan Garrett, Mark D Hicar

**Affiliations:** 1 University at Buffalo, Buffalo, New York; 2 University of Washington, Seattle, Washington

## Abstract

**Background:**

Recent data supports that improved qualitative antibody responses correlate with elite controllers (EC) of HIV. As ADCC has been associated with protection in vaccine studies, thorough exploration of antibodies that facilitate ADCC is warranted. In studies on monoclonal antibodies from long-term non-progressors (LTNPs), our laboratory has previously described highly mutated antibodies against a complex conformational epitope with contributions from both gp41 heptad repeat regions. Despite using the VH1-02 gene segment, known to contribute to some of the broadest neutralizing antibodies against HIV, members of these antibodies, termed group 76C antibodies, did not exhibit broad neutralization.

**Methods:**

Our goal was to characterize the non-neutralizing functions of antibodies of group 76C, to assess targeting of the epitope in various clinical presentations, and to assess the development of these antibodies by comparison to their predicted common ancestor. Serum samples were obtained from HIV+ clinical groups: EC, LTNP, stable CD4 counts on therapy, and those off therapy.

**Results:**

In antibody/serum competition assays, comparison to VRC01 which also uses VH1-02, showed that antibodies targeting the 76C group epitope were enriched in LTNPs. We then show recombinant antibodies of 76C members 6F5 and 6F11 both have robust ADCC activity, despite their sequence disparity. Sequence analysis predicted the common ancestor of this clonal group would utilize the germline non-mutated variable gene. We produced a recombinant ancestor Ab (76Canc) with a heavy chain utilizing the germline variable gene sequence paired to the 6F5 light chain. 76Canc binds HIV envelope constructs near the original group C epitope. 76Canc also shows comparable ADCC to 6F5 and 6F11 on both clade B and C constructs.

Common ancestor antibodies maintain function and these types of antibodies correlate to a non-progressive clinical state.

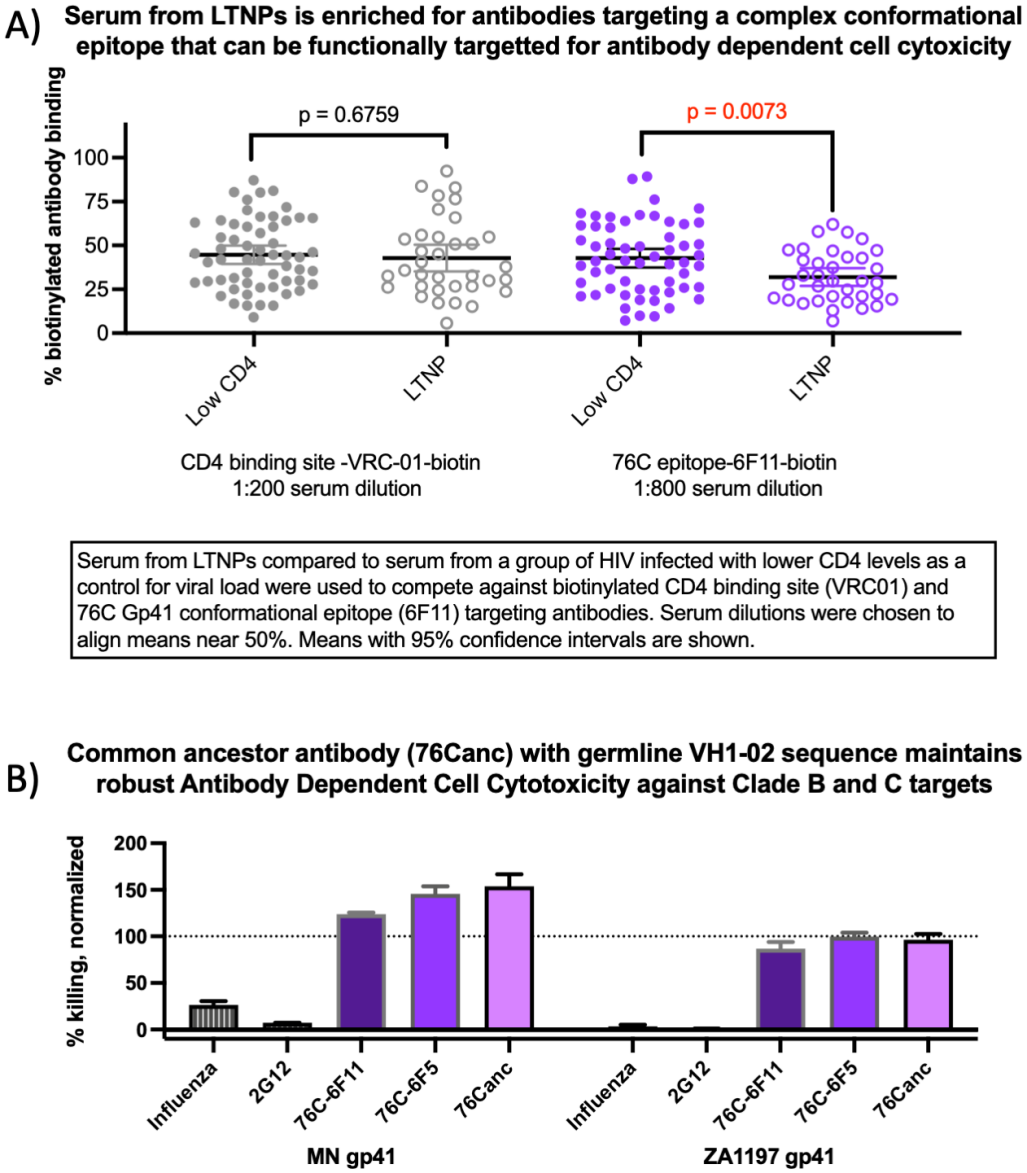

(A) Serum from long-term non-progressors (LTNPs) compared to serum from a group of HIV infected with lower CD4 levels as a control for viral load were used to compete against biotinylated CD4 binding site (VRC01) and 76C Gp41 conformational epitope (6F11) targeting antibodies. Serum dilutions were chosen to align means near 50%. Means with 95% confidence intervals are shown. (B) Monoclonal antibody 76Canc was created using the germline sequence of the heavy chain variable region with the CDR3 and light chain of 76C member. Antibody dependent cell cytotoxicity flow cytometric based assays were performed using gp41 proteins from clade B (MN) and clade C (ZA1197).

**Conclusion:**

Certain antibodies present early on in infection may contribute to overall clinical course. Variable gene germline sequences that support functional activity against HIV could be targeted in vaccine regimens.

**Disclosures:**

**All Authors**: No reported disclosures

